# Assessment and analysis of content and quality of children's influenza vaccine information on BiliBili, Douyin, and Xiaohongshu

**DOI:** 10.3389/fdgth.2025.1591347

**Published:** 2026-01-30

**Authors:** Shanwei Jiang, Hui Sun, Xiaowei Chen

**Affiliations:** Yantaishan Hospital, East Campus, Yantai, China

**Keywords:** influenza vaccine, child, information quality, social media, short videos

## Abstract

**Background:**

Each year, influenza vaccines play a vital role in preventing millions of illnesses and reducing flu-related healthcare visits. Short video platforms (including Douyin, BiliBili, and Xiaohongshu) are powerful vehicles for information sharing and are saturated with videos about children's influenza vaccines. Nevertheless, the quality of these videos remains undetermined.

**Purpose and objectives:**

This study aims to assess the quality and reliability of videos addressing children's influenza vaccines on three short video platforms: Douyin, BiliBili, and Xiaohongshu.

**Methods:**

Using a cross-sectional survey design, this study examined three short video platforms (all mainland China versions). In February 2025, the research team searched Douyin, BiliBili, and Xiaohongshu for the keyword “children's influenza vaccine,” selecting 300 videos (100 per platform) for analysis. We extracted basic video information, coded the content, and identified each video's source. Two independent reviewers then evaluated video quality using the American Medical Association (JAMA) benchmarks, the modified DISCERN (mDISCERN) criteria, and the Global Quality Score (GQS).

**Results:**

A detailed analysis of 300 videos revealed that on Douyin and Xiaohongshu, most videos were created by professionals and lay users, whereas on BiliBili, most were uploaded by non-profit organizations. Douyin stood out in user engagement, as its videos received significantly higher numbers of likes, comments, favorites, and shares than those on BiliBili and Xiaohongshu (*p* < 0.001). Furthermore, we observed a strong positive correlation between overall quality scores and comment volume (Spearman *ρ* = 0.90, *p* < 0.001); correlations with likes (*ρ* = 0.77) and favorites (*ρ* = 0.73) were moderate but still significant (*p* < 0.001). Score differences also emerged based on source type (*p* < 0.001). Videos published by health professionals were rated highest, ordinary-user videos received lower ratings, and those from news agencies and non-profit organizations fell in between. In terms of quality ratings, there were no statistically significant differences among the GQS, JAMA, and mDISCERN scoring systems.

**Conclusion:**

These findings suggest that Douyin, BiliBili, and Xiaohongshu offer moderately rated scientific content regarding children's influenza vaccines. Viewers should exercise caution when watching related videos on these platforms. Moving forward, both the platforms and content creators must strive to improve video quality and reliability to boost vaccination rates. These efforts have important implications for clinical practice, offering new perspectives for health education interventions and better promoting public awareness of vaccination's significance—ultimately contributing positively to public health.

## 1 Introduction

Influenza, commonly known as the flu, is an acute respiratory infection caused by the influenza virus and poses a serious threat to human health. While everyone is potentially at risk, certain groups—pregnant women, infants and toddlers, older adults, and those with chronic conditions—are especially vulnerable to more severe illness once infected. In children, clinical signs of influenza vary widely, ranging from asymptomatic presentations to severe cases and, in rare instances, even death. Influenza-like illness is typically defined as fever accompanied by cough or sore throat. Notably, during flu season, the possibility of influenza infection cannot be ruled out solely by the absence of fever, particularly when respiratory symptoms are present. Among children aged 15 and older, asymptomatic infection occurs in about 26% of cases, while 6.6% of younger children may also remain asymptomatic. In children under 4 years old, around 40% may develop gastrointestinal symptoms, such as vomiting and diarrhea. Older children are more likely than younger children to experience headaches, with other common symptoms including general discomfort, fatigue, and muscle aches. An estimated 28,000 children under 18 die each year from influenza-related lower respiratory tract infections worldwide, with the majority of these deaths occurring in children under four (1–3).

Children play a pivotal role in spreading influenza because they often carry higher viral loads, exhibit greater infectiousness, and shed the virus for longer periods. Their frequent movement between school and home also accelerates the spread of flu. In addition to posing a direct health threat, childhood influenza incurs a notable socioeconomic burden on families. Nearly all school-aged children with the flu miss at least 1 day of school, and their total missed school days exceed those associated with other common pediatric acute respiratory infections like respiratory syncytial virus, human metapneumovirus, parainfluenza virus, and coronavirus. Furthermore, about half of parents of children with influenza miss at least one workday to provide care, and many must hire additional help. Over the course of illness, children with flu visit healthcare providers at least once, inflating family medical costs and increasing the strain on healthcare systems (1). Research indicates that getting a flu shot significantly reduces antibiotic usage, general practitioner visits, fever-related illnesses, sick leave, and flu-related trips to the emergency department or admission to the ICU. Remarkably, when children in a household receive the influenza vaccine, the risk of unvaccinated adults contracting the flu is halved. Compared with other pediatric vaccinations, the flu vaccine offers high cost-effectiveness, largely because of its notable direct and indirect benefits (3).

The World Health Organization (WHO) strongly recommends routine flu vaccination for children between 6 and 59 months of age, and China's CDC likewise designates this group as a priority for immunization (4). Studies indicate that vaccination prevents 16% of flu-related hospitalizations among children aged 5–17, and 28% among those aged 6 months to 4 years. Moreover, flu shots not only mitigate disease severity but also substantially reduce mortality from influenza—by 51% in high-risk children and by 65% in otherwise healthy children (5). Despite the proven safety and effectiveness of the influenza vaccine, global uptake remains low. Studies show that flu vaccination coverage in China is just 3.84%, while in the United States, only 38%–62% of children under 18 are vaccinated (6). Barriers to widespread immunization among children include limited public awareness, a lack of healthcare provider recommendations, and worries about vaccine safety (7). According to the 2024 annual survey of short video usage, more than one billion people worldwide use these platforms, with an average daily viewing time of 85 min. This widespread penetration of short videos—paired with the public's growing tendency to seek health-related guidance from social media (rather than traditional channels like healthcare providers)—brings dual implications for children's influenza vaccine promotion: on one hand, it offers an efficient pathway to deliver evidence-based information to a broad audience (e.g., parents learning about vaccination schedules or safety data); on the other hand, it raises risks of misinformation dissemination—given that unsubstantiated claims about vaccine side effects or ineffectiveness can spread rapidly across these platforms, directly undermining parental trust in influenza vaccination. As new media channels proliferate, increasing numbers of people turn to social media for health information. However, verifying the accuracy of content on these platforms can be challenging, creating opportunities for public misinformation. While the internet is a common source of vaccine information—encompassing reputable official channels alongside considerable amounts of unreliable or false content—this varied information landscape can diminish the impact of health campaigns. Although social media has become a critical source of health information, its inconsistent quality may further erode public understanding of and willingness to receive flu vaccinations (8). This study seeks to examine and assess the quality of videos about children's influenza vaccines on three short video platforms operating in mainland China (Douyin, BiliBili, Xiaohongshu). By doing so, it aims to foster a more comprehensive and responsible public understanding of flu vaccination and to encourage wider access to trustworthy information on influenza.

## 2 Methods

### 2.1 Platform identification and justification

This study confines its scope to three short-video platforms (Douyin, BiliBili, and Xiaohongshu)—all legally accessible and culturally dominant within mainland China. The platforms selected and the rationale for their selection, along with detailed access methods, are elaborated below.

#### 2.1.1 Douyin (mainland China version)

Operated by ByteDance, Douyin is the most widely adopted short-video application on the mainland. Its core demographic comprises parents aged 25–45. The platform's content ecosystem is tightly attuned to Chinese cultural norms and public-health priorities—for example, vaccination explainers that explicitly reference China CDC schedules and protocols. Operationally, Douyin is fully compliant with PRC internet regulations, including the Measures for the Administration of Internet Health Information Services, ensuring that vaccine-related content is consistent with national guidelines.

#### 2.1.2 Bilibili (mainland China version)

BiliBili is a video-sharing community whose primary user cohort falls between 18 and 30 years old; this cohort includes tertiary-level medical students and newly minted parents. The platform privileges science communication and granular, evidence-based explanations, aligning with the demand among younger caregivers for in-depth knowledge about influenza vaccination.

#### 2.1.3 Xiaohongshu (mainland China version)

Xiaohongshu is a lifestyle-sharing network where approximately most of users are women, most aged 25–35 and many of whom are mothers. The platform's dominant modality is experiential storytelling (e.g., “Our child's vaccination day”), which dovetails with Chinese parents' desire for practical, real-world insights into the vaccination process.

#### 2.1.4 Exclusion of TikTok

TikTok is neither legally nor technically accessible in mainland China. Its user base skews toward teenagers and young adults outside China, and its content is governed by markedly different regulatory standards—particularly looser health-information moderation policies. Furthermore, its algorithmic logic favors globally trending topics rather than China-specific health concerns.

### 2.2 Access methods

#### 2.2.1 Region

Restricted to mainland China to ensure alignment with the target audience (Chinese parents seeking children's influenza vaccine information) and compliance with local regulatory frameworks.

#### 2.2.2 Device

A brand-new, unconfigured smartphone (Xiaomi 14) with no pre-installed third-party applications, no residual user behavior data (browsing history, app usage records), and disabled personalized permission settings (location access, device information tracking).

#### 2.2.3 Account type

A newly registered regular user account (no prior activity, no professional certification, no enterprise verification) created on February 20, 2025, using a mainland China mobile phone number for real-name authentication (consistent with PRC internet regulations).

### 2.3 Sample size and power

We conducted an *a priori* power analysis to justify the sample size for both the three-platform comparison and the correlation analyses.

#### 2.3.1 Between-platform comparison

To approximate the required sample size for the Kruskal–Wallis test, we used a one-way ANOVA on ranks and expressed the effect size as Cohen's f. With a two-sided *α* = 0.05% and 80% power, a total sample of *n* = 300 videos (100 per platform) yields at least 80% power to detect a small-to-moderate effect of *f* ≈ 0.18. For comparison, detecting effect sizes of *f* = 0.20, *f* = 0.25, and *f* = 0.30 would require total samples of approximately 246 (≈82 per platform), 156 (≈52 per platform), and 111 (≈37 per platform), respectively.

#### 2.3.2 Correlation analyses

For the associations between video-quality scores (GQS, JAMA, mDISCERN) and engagement metrics, we used Fisher's *z* transformation applied to Pearson correlations, which closely approximates Spearman correlations in moderately large samples. With *α* = 0.05 (two-sided) and 80% power, a total sample of *n* = 300 can detect correlations as small as *r* ≈ 0.16. Because the observed correlations in our data were substantially larger, the study is adequately powered to detect the reported effects.

### 2.4 Search strategy and data processing

To reduce bias when analyzing newly uploaded videos, the following standardized protocols were implemented: To reduce bias when analyzing newly uploaded videos, the following standardized protocols were implemented based on the access methods detailed in Section 2.2.

1. All searches were restricted to the Chinese mainland. This is because Douyin, BiliBili, and Xiaohongshu have their core operations and regulatory frameworks centered in the Chinese mainland, with their key user demographics, content libraries, and algorithmic recommendation logic all tailored to this region. Limiting searches to the Chinese mainland prevents content restrictions or algorithmic biases that may occur with cross-regional access, ensuring the retrieved videos match the actual information exposure scenarios of the target audience (Chinese parents seeking information on children's influenza vaccines).
2. On February 20, 2025, researchers created a separate new account for each of the three platforms (Douyin, BiliBili and Xiaohongshu). Search results were collected based on the platforms' default algorithms without applying any filters, ensuring the objectivity of sample selection for initial analysis. These new accounts had no prior user activities (e.g., search history, likes, follows) or qualification certifications (e.g., medical professional certification, corporate verification), which prevented personalized recommendation algorithms from biasing the search results.

**Figure 1:**
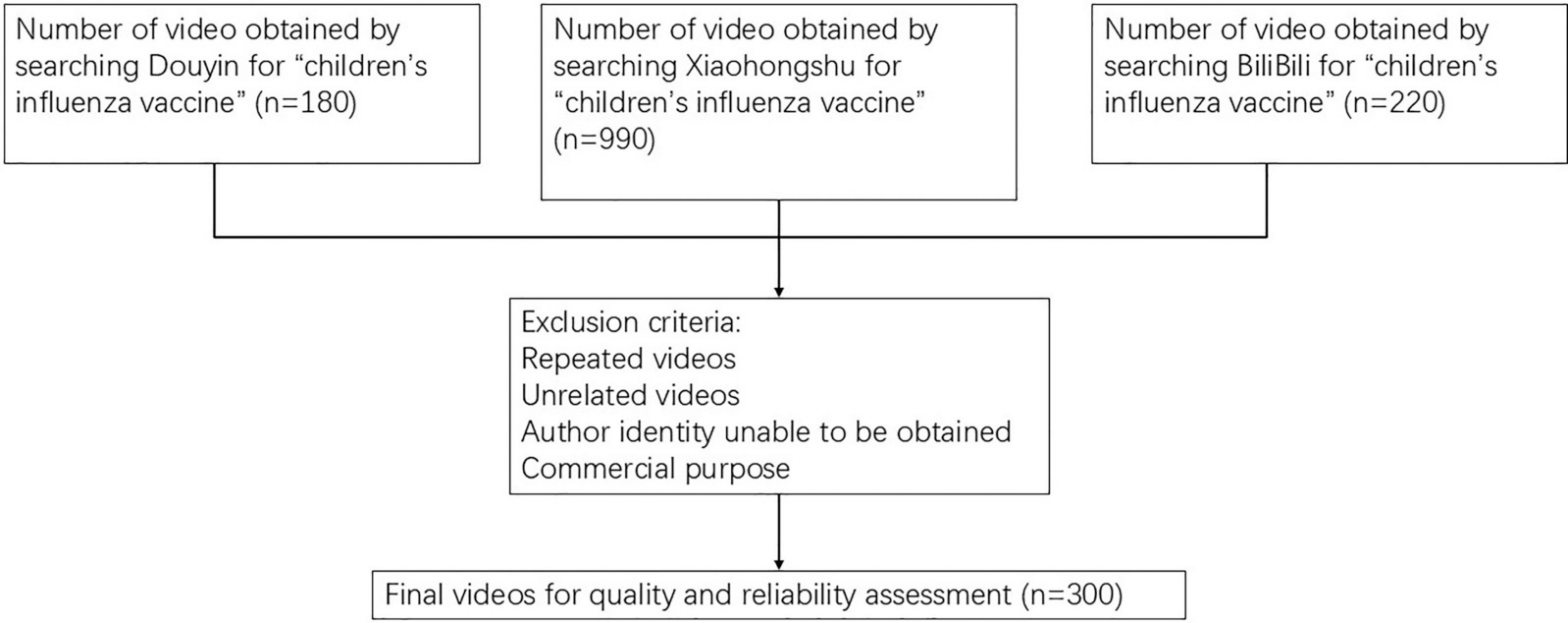
Flow-chart depicting the selection process of videos.

Subsequently, searches were conducted on the three platforms using the keyword “children's influenza vaccine,” with results sorted by the platforms' default methods. All retrieved videos were evaluated against predefined inclusion and exclusion criteria specific to children's influenza vaccine content: irrelevant videos (e.g., those about adult influenza vaccines or general children's health knowledge) were excluded, and only content directly related to the topic was retained. Finally, the first 100 eligible Chinese-language videos (Figure 1) were selected for comprehensive review.

**Figure 2:**
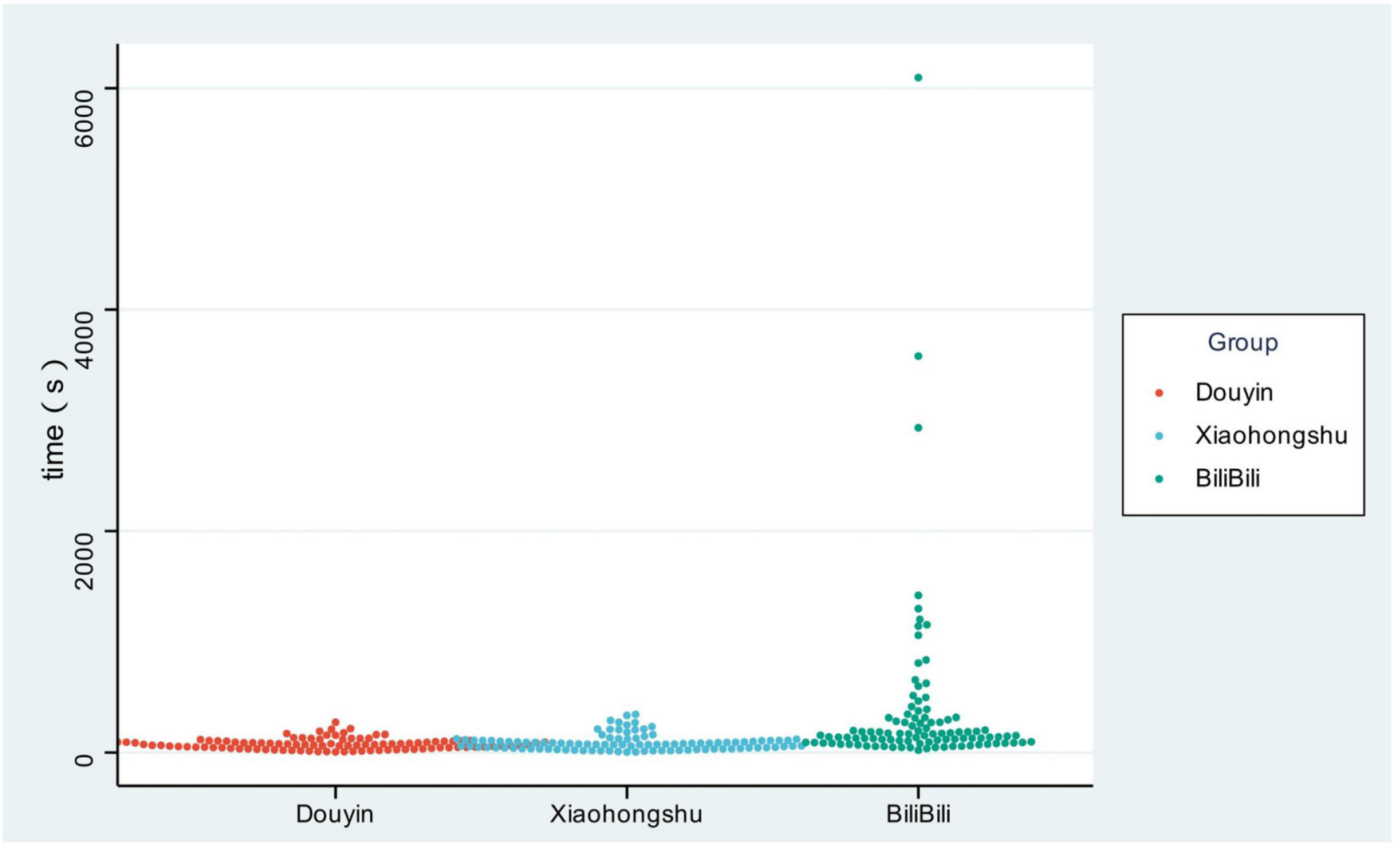
The playback time of children's influenza vaccine -related videos on the three different platforms.

For each video, the research team collected detailed metadata, including title, duration, days since upload, view count, likes, shares, comments, and video source, to support subsequent analysis. Videos were then categorized by uploader type: healthcare professionals, general users, news organizations, and non-profit groups. Each video was evaluated using the following standardized tools.

1. Commenting behavior operational definition.

To address the multidimensional nature of user engagement with video content, this study defines “commenting behavior” as encompassing two distinct but complementary dimensions: comment quantity and comment sentiment, with clear operational criteria to ensure consistency and reproducibility.

#### 2.4.1 Comment quantity

This refers to the total number of original user comments displayed on each video at the time of data collection (February 25–28, 2025). It is treated as a continuous variable, with raw counts extracted directly from the platform interfaces (Douyin, BiliBili, and Xiaohongshu) to reflect the overall level of user participation in discussing the video content.

#### 2.4.2 Comment sentiment

This dimension categorizes the emotional tone of user comments into three mutually exclusive categories (categorical variable), based on a standardized codebook developed for this study (available from the corresponding author upon request). The specific classification criteria and illustrative examples are as follows:

- Positive sentiment: Comments expressing support for children's influenza vaccination (e.g., “We took our kid for the flu shot last week, and it went smoothly—highly recommend!”) or recognition of the video's information accuracy (e.g., “This video clearly explains the vaccination schedule, which matches what our pediatrician told us”).
- Negative sentiment: Comments voicing vaccine hesitancy or skepticism (e.g., “I heard the flu vaccine has many side effects, so I’m hesitant to let my kid get it”) or pointing out inaccuracies in the video content (e.g., “The video says the vaccine is for kids over 6 months, but my doctor said some brands are for 3 months and above—this is misleading”).
- Neutral sentiment: Comments posing factual questions unrelated to emotional stance (e.g., “Where can I make an appointment for the children's flu vaccine?”) or remarks irrelevant to the core topic (e.g., “The background music in this video is nice”).

### 2.5 Video quality assessments

The reliability of the videos was assessed using the criteria from the Journal of the American Medical Association (JAMA). Each video could accumulate up to four points, with one point awarded for each criterion met; a higher score indicated greater reliability (9). The Global Quality Score (GQS) utilized a five-tiered scale to evaluate the overall quality of information and its applicability for patients, with higher levels reflecting better quality, coherence, comprehensiveness, and utility for patients (10). The modified DISCERN instrument, a widely used standard for assessing the quality of consumer health information, was also applied to video content. This instrument focuses on the content's accessibility, impartiality, and source traceability. Scoring involved five questions, with affirmative responses scored as 1 and negative responses as 0, resulting in a range from 0 to 5, where higher scores indicated superior reliability and quality (11).

### 2.6 Data recording and reliability assessment

All data were recorded in Excel (Microsoft Corporation). To minimize potential scoring bias, the list of video links was randomized. Basic video information and content quality were documented and assessed independently by two reviewers. Inter-rater reliability was calculated using Cohen's *κ*, yielding values > 0.8 for all evaluated items, indicating excellent agreement. Any discrepancies were resolved by a third investigator.

### 2.7 Statistical analysis

SPSS27.0 statistical software was used to analyze the data. Median and interquartile range (IQR) were used for non-normally distributed data, while frequency and percentage were used for categorical data. The Kruskal–Wallis test was used for between-group comparisons of non-normally distributed quantitative variables. The correlations between different datasets were analyzed using Spearman correlation analysis. Statistical significance was set at *p* < 0.05.

## 3 Results

### 3.1 General information

To systematically characterize the basic attributes of the 300 included videos (100 per platform), we first extracted and analyzed key metrics, including duration, user engagement (likes, comments, favorites, shares), and quality scores (GQS, JAMA, mDISCERN). The detailed characteristics of videos across Douyin, BiliBili, and Xiaohongshu are summarized in Table 1.

In terms of video duration (a core indicator reflecting content form), significant differences were observed among the three platforms (*p* < 0.001). BiliBili videos had the longest median duration at 153 s [interquartile range (IQR): 90.25–308 s], nearly twice that of Douyin (72 s, IQR: 44–101.75 s) and Xiaohongshu (75.5 s, IQR: 35.5–109 s). Notably, BiliBili also exhibited the largest IQR for duration, indicating greater variability in content length—ranging from short introductory clips (≈90 s) to long-form technical discussions (≈300 s). In contrast, Douyin and Xiaohongshu had narrower IQRs, reflecting more standardized, concise content that aligned with their users' preference for fragmented information consumption.

For user engagement metrics (used as a proxy for information dissemination efficiency), Douyin demonstrated a clear advantage across all dimensions (*p* < 0.001). The median number of likes on Douyin was 567.5 (IQR: 84.75–2135.5), which was substantially higher than BiliBili's 9 (IQR: 2–69) and Xiaohongshu's 18.5 (IQR: 5.25–136). Similar trends were observed for comments (Douyin: 32.5, IQR: 6.25–462.75; BiliBili: 0, IQR: 0–1; Xiaohongshu: 3.5, IQR: 1–26), favorites (Douyin: 193.5, IQR: 20.75–817; BiliBili: 7, IQR: 1.25–36.75; Xiaohongshu: 6, IQR: 1.25–40.75), and shares (Douyin: 361, IQR: 34–2253; BiliBili: 6.5, IQR: 0–32.5; Xiaohongshu: 7, IQR: 7–28.25). This high engagement on Douyin may be attributed to its content positioning: practical, life-oriented videos (e.g., vaccination appointment guides, post-vaccination care tips) that directly address parents' urgent needs, thereby encouraging active interaction.

Regarding video quality (assessed via GQS, JAMA, and modified DISCERN), no statistically significant differences were found among the three platforms (all *p* > 0.05). The median GQS scores were 3 (IQR: 2–3.75) for Douyin, 3 (IQR: 2–3) for BiliBili, and 3 (IQR: 2–4) for Xiaohongshu; JAMA scores were 3 (IQR: 2–3), 2.5 (IQR: 2–3), and 2.5 (IQR: 1–4), respectively; and modified DISCERN scores were 2 (IQR: 2–3) across all three platforms. These results indicate that despite differences in content length and user engagement among the three platforms, the overall quality of children's influenza vaccine information is comparable across them, with most videos classified as “moderate quality.”

After presenting the quantitative characteristics of videos across platforms in Table 1, we further visualized the differences in video playback time using Figure 2. As shown in Figure 2, the distribution of playback time—consistent with the quantitative data in Table 1—varied significantly among the three platforms (*p* < 0.001). BiliBili videos exhibited the longest duration, with most ranging from approximately 90 seconds to over 300 s, which aligns with its core positioning of “in-depth science communication”—targeting users (e.g., medical students, young caregivers) who demand detailed explanations of complex topics such as influenza vaccine mechanisms. In contrast, Douyin and Xiaohongshu videos were relatively concise, with most falling between 35 and 110 s. This shorter duration is consistent with the platforms' user demographics: Douyin's primary audience (parents aged 25–45) prefers fragmented, practical information (e.g., vaccination appointment guides), while Xiaohongshu's core users (mothers aged 25–35) tend to engage with scenario-based experience sharing (e.g., “My child's vaccination day”), both of which prioritize efficient information delivery.

Notably, the visual trend in Figure 2 also echoes the IQR data in Table 1: BiliBili had the largest IQR (90.25–308 s), indicating greater variability in video duration—likely due to its tolerance for both short introductory content and long-form technical discussions. Meanwhile, Douyin (44–101.75 s) and Xiaohongshu (35.5–109 s) showed narrower IQR ranges, reflecting more standardized content length tailored to their users' preference for concise, focused information. This platform-specific duration pattern not only reflects differences in user needs but also provides a foundation for analyzing how content form influences information dissemination efficiency in subsequent sections.

**Table 1:** Characteristics of the videos across sources.

Parameters	Douyin (*N* = 100)	BiliBili (*N* = 100)	Xiaohongshu (*N* = 100)	*p*-value
Duration, median (IQR) (s)	72 (44–101.75)	153 (90.25–308)	75.5 (35.5–109)	<0.001
Likes, median (IQR)	567.5 (84.75–2,135.5)	9 (2–69)	18.5 (5.25–136)	<0.001
Comments, median (IQR)	32.5 (6.25–462.75)	0 (0–1)	3.5 (1–26)	<0.001
Collections, median (IQR)	193.5 (20.75–817)	7 (1.25–36.75)	6 (1.25–40.75)	<0.001
Forwarding volume, median (IQR)	361 (34–2,253)	6.5 (0–32.5)	7 (7–28.25)	<0.001
GQS score, median (IQR)	3 (2–3.75)	3 (2–3)	3 (2–4)	0.283
JAMA score, median (IQR)	3 (2–3)	2.5 (2–3)	2.5 (1–4)	0.560
Modified DISCERN score, median (IQR)	2 (2–3)	2 (2–3)	2 (1–3)	0.522

In terms of video quality, the Global Quality Score (GQS) IQRs were 3 (2–3.75) on Douyin, 3 (2–3) on BiliBili, and 3 (2–4) on Xiaohongshu, with no statistically significant differences (*p* = 0.283). The JAMA scores—3 (2–3) on Douyin, 2.5 (2–3) on BiliBili, and 2.5 (1–4) on Xiaohongshu—also showed no significant variation (*p* = 0.560). Likewise, the mDISCERN scores of 2 (2–3) on Douyin, 2 (2–3) on BiliBili, and 2 (1–3) on Xiaohongshu revealed no meaningful differences (*p* = 0.522). Taken together, these findings suggest that overall video quality was comparable across all three platforms.

### 3.2 Content analysis of online videos

**Figure 3:**
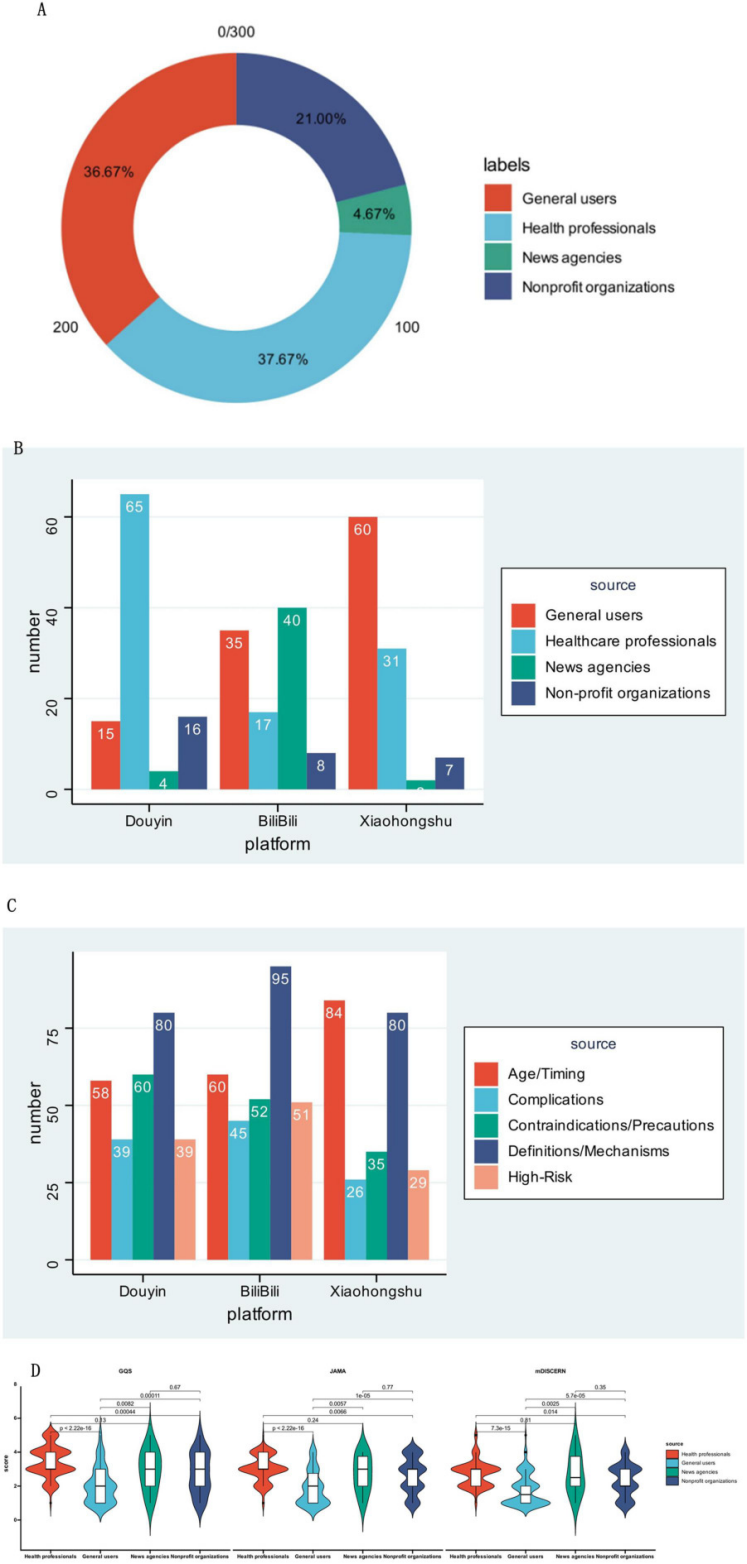
The source on three different platforms: **(A)** doughnut chart shows the sources of all included videos. **(B)** Bar chart shows the sources of videos from Douyin, BiliBili and Xiaohongshu. **(C)** Bar chart shows the content of videos from Douyin, BiliBili and Xiaohongshu **(D)** The violin plot shows the scores of videos from different sources.

Table 2 classifies video publishers into four groups: general users, healthcare professionals, news agencies, and non-profit organizations. Among these, healthcare professionals contributed the largest share of videos (37.67%), followed by general users (36.67%), highlighting their pivotal roles in disseminating vaccine information. Non-profits accounted for 21.00%, and news outlets 4.67% (Figure 3A). By platform, healthcare professionals led on Douyin (65%, *n* = 65), with general users at 15% (*n* = 15), news agencies at 4% (*n* = 4), and non-profits at 16% (*n* = 16). On BiliBili, non-profits were dominant (40%, *n* = 40), followed by general users (35%, *n* = 35), healthcare professionals (17%, *n* = 17), and news agencies (8%, *n* = 8). On Xiaohongshu, general users formed the majority (60%, *n* = 60), followed by healthcare professionals (31%, *n* = 31), news agencies (2%, *n* = 2), and non-profits (7%, *n* = 7) (Figure 3B). Although their sources differed, publishers across all three platforms overwhelmingly demonstrated positive attitudes toward flu vaccination (100% on Douyin, 99% on BiliBili, and 99% on Xiaohongshu). However, when looking at popular comments, Douyin had the highest rate of positive sentiment (48%), while BiliBili and Xiaohongshu trailed behind at 21% and 19%, respectively. This variation in comment sentiment could be further explained by platform-specific content characteristics. Douyin's high positive comment rate (48%) was closely linked to its high proportion of healthcare professional content (65%)—videos such as vaccination appointment guides and post-vaccination care tips directly addressed parents’ practical needs, reducing skepticism. BiliBili's lower positive rate (21%) corresponded to its focus on in-depth mechanism discussions (95 videos on influenza vaccine mechanisms, the highest among the three platforms), where comments often involved technical debates (e.g., comparing vaccine types) rather than emotional support. Xiaohongshu's 19% positive rate aligned with its experiential content focus (84 videos on recommended vaccination age/timing, the most across platforms), as personal stories tended to elicit more neutral feedback (e.g., “I’ll check with my doctor first”) rather than strong positive sentiment. Video content was grouped into five categories: definitions/mechanisms of action, recommended vaccination age/time, high-risk groups, complications, and contraindications/precautions. Definitions/mechanisms and vaccination age/time were the most frequently covered topics, whereas complications and high-risk populations received comparatively less attention. Specifically, BiliBili contained the highest number of videos in the definitions/mechanisms category (95 videos), while Xiaohongshu featured the most in the age/time category (84 videos) (Figure 3C). Violin-plot analyses of GQS, JAMA, and mDISCERN scores revealed that videos uploaded by healthcare professionals received the most favorable assessments, general users the least, and news agencies and non-profits ranked in between. These differences were statistically significant across sources (Figure 3D).

**Table 2:** Comparison of source on three different platforms.

Video source	Douyin (*N* = 100)	BiliBili (*N* = 100)	Xiaohongshu (*N* = 100)
Health professionals (*N*) (%)	65 (65%)	17 (17%)	31 (31%)
General users (*N*) (%)	15 (15%)	35 (35%)	60 (60%)
News agencies (*N*) (%)	4 (4%)	8 (8%)	2 (2%)
Nonprofit organizations (*N*) (%)	16 (16%)	40 (40%)	7 (7%)
Actively participate in vaccination			
Video publisher (*N*) (%)	100 (100%)	99 (99%)	99 (99%)
Top Comments (*N*) (%)	48 (48%)	21 (21%)	19 (19%)

### 3.3 Correlation analysis

**Figure 4:**
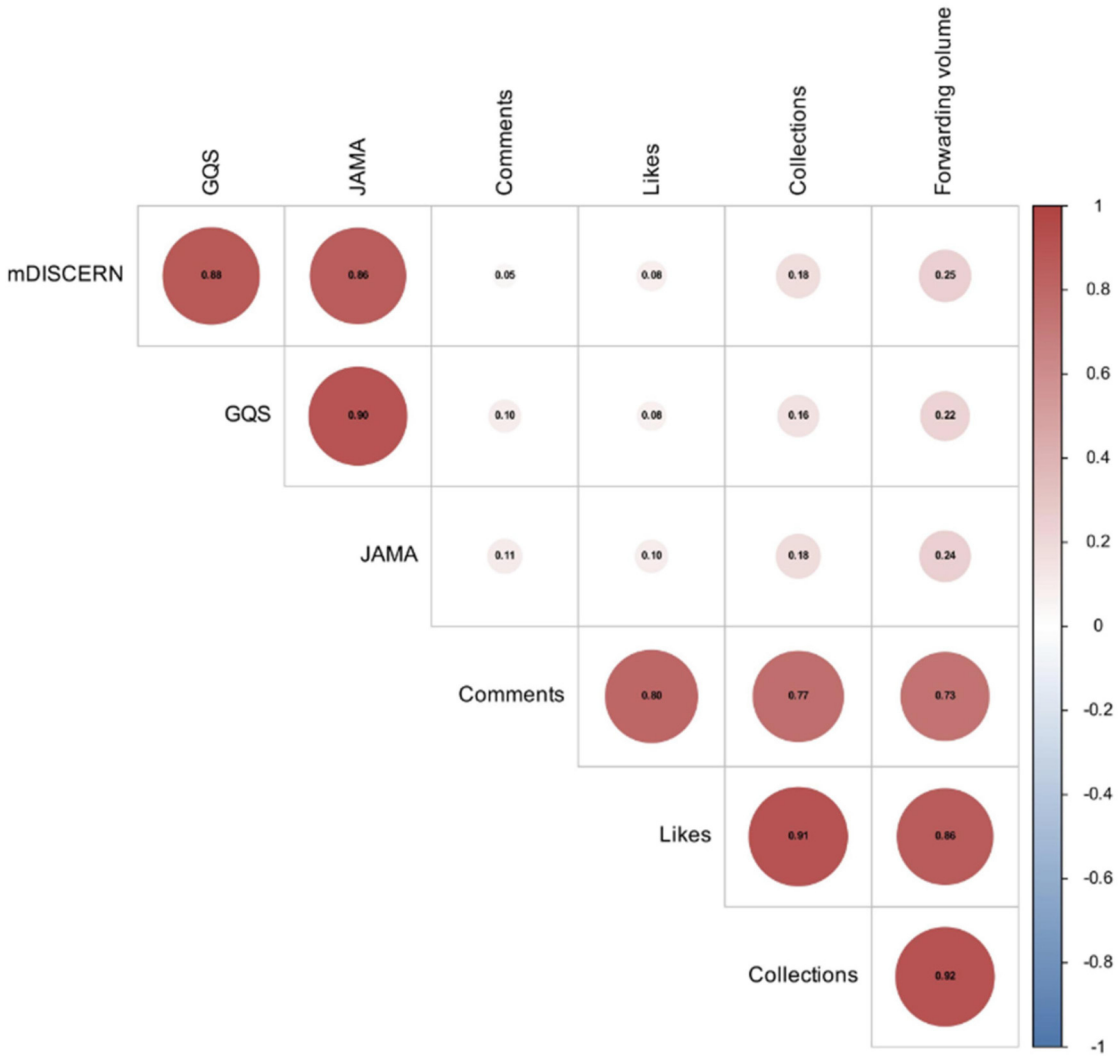
Spearman correlation analysis corrplot.

Spearman's correlation analysis revealed a coefficient of 0.90 between GQS and JAMA, with mDISCERN showing correlations of 0.88 and 0.86 when compared to GQS and JAMA, respectively. Among engagement metrics, the strongest positive correlation emerged between the scoring systems and comment counts. Notably, GQS's correlation with comments was 0.90, reflecting a very robust association, and mDISCERN's correlation with comments was 0.80, also indicating a strong link. Although correlations between the scoring systems and favorites/likes were somewhat weaker, they remained positively aligned. Moreover, comments, favorites, and likes were strongly intercorrelated: favorites and likes exhibited a coefficient of 0.91, while comments and likes correlated at 0.80, and comments and favorites at 0.77. These findings underscore that video scores are tightly coupled with user comment volume. Although likes and favorites correlate slightly less with the scoring systems, they still show strong positive interrelationships, making them valuable complementary indicators of a video's popularity (Figure 4).

## 4 Discussion

Influenza is a serious public health concern, contributing to high rates of illness and death on a global scale. According to data, nearly one million children under age five require hospitalization each year for flu. In the United States specifically, 8%–10% of children develop symptomatic influenza annually, resulting in 140,000–710,000 hospital stays and 12,000–52,000 deaths. Flu incidence varies by season, and unvaccinated children face a substantially higher infection rate—20%—compared to 10% among vaccinated peers (5). The American Academy of Pediatrics strongly advocates annual flu vaccinations for all children over 6 months of age who have no medical contraindications. Nevertheless, vaccination rates remain below desired levels. Key factors include limited public awareness of flu and its vaccine, varied attitudes toward immunization within families, an absence of clear government directives, worries about vaccine quality, and practical barriers such as supply shortages (12, 13). With the emergence of new media, social platforms have become a leading source of personal health information and advice. In the United States, understanding how social media influences vaccine confidence a top priority in immunization research is now. There is an urgent need for studies that directly inform evidence-based strategies for sharing vaccine information on social networks. Although negative vaccination messages online often lack credibility, they can still sow confusion and doubt among parents weighing the decision to immunize their children. Consequently, ensuring that vaccine-related content on social media is accurate and trustworthy is crucial for maintaining public trust and boosting vaccination rates. Research indicates that transparent sharing of factual, evidence-based information—such as cost-effectiveness data—is highly effective for easing concerns among target audiences, including those worried about vaccine safety. By openly debating vaccine safety and actively promoting immunization benefits within diverse communities, public health campaigns can markedly increase individuals' readiness to receive flu shots. Such evidence-driven communication strategies not only foster public trust in vaccines but also encourage decisions that benefit both personal and community health (14, 15).

Douyin, BiliBili, and Xiaohongshu are all legally compliant, mainstream short-video/content platforms in mainland China with substantial user bases—their monthly active users (MAU) had reached 766 million, 120 million, and 100 million respectively. These platforms cover diverse age groups, genders, and regions, and our analysis of 300 videos (100 per platform) revealed distinct characteristics shaped by their audience demographics and regulatory environments, directly influencing content credibility, user engagement, and information dissemination reliability: Douyin, with its core audience of 25–45-year-old parents, enforces strict medical content review (aligned with China's Internet Health Information Services regulations), fostering a high proportion of professional content (65% from healthcare professionals) and strong user engagement (median likes: 567.5, comments: 32.5)—its varied, life-oriented content (e.g., vaccination appointment guides, post-vaccination care tips) resonates closely with parents' practical needs. By contrast, BiliBili targets 18–30-year-old users (including medical students and young caregivers) and prioritizes in-depth science communication (e.g., 95 videos on influenza vaccine mechanisms, the highest among the three platforms); however, its looser review of non-certified content introduces risks of unsubstantiated claims, and its users' “watch-without-interacting” behavior results in low engagement (median likes: 9, comments: 0). Xiaohongshu, dominated by 25–35-year-old mothers, centers on personal experience-sharing (e.g., 84 videos on vaccination age/timing, the most across platforms) with moderate content quality; yet its weak oversight of medical advice in comment sections (e.g., unsubstantiated “side effect warnings” from ordinary users) and high proportion of non-professional uploads (60%) limit information reliability. Additionally, unified access conditions, namely the use of a new device, new accounts, and unfiltered searches—minimize biases to the greatest extent, ensuring sample objectivity and comparability.

Notably, these platform-specific traits not only shape how health-related videos are produced and presented but also highlight the need to tailor health communication strategies to each platform's unique strengths and limitations. Differences in audiences and regulatory practices further significantly impact the interpretation of study findings: unique user characteristics (e.g., Douyin users prioritizing practical vaccination guides, BiliBili users focusing on vaccine mechanism popularization, Xiaohongshu users preferring personal experience content) and slight variations in review standards (e.g., Douyin's stricter medical content review vs. BiliBili's greater tolerance for popular science) lead to subtle differences in content quality scores and information reliability across platforms. Thus, the communication effects and quality characteristics of children's influenza vaccine information must be comprehensively interpreted based on each platform's unique attributes.

Douyin excels in user engagement, with significantly higher likes, comments, favorites, and shares than BiliBili and Xiaohongshu. BiliBili, by contrast, has lower engagement but features longer videos—these longer formats potentially enable more in-depth discussions and detailed explanations of topics like influenza vaccine mechanisms. Xiaohongshu's engagement levels fall between the two platforms. Notably, video quality scores correlate strongly with comment quantity (Spearman *ρ* = 0.90, *p* < 0.001), while likes and favorites, though moderately correlated (*ρ* = 0.77 and 0.73 respectively, *p* < 0.001), remain positively linked; together, these metrics serve as useful joint indicators of a video's popularity. These platform-specific engagement variations are critical for shaping effective health communication strategies and enhancing the dissemination of children's influenza vaccine information across social media.

In terms of content, definitions/mechanisms of the influenza vaccine and recommended vaccination age/timing are the most frequently covered topics—reflecting high user demand for basic vaccine facts—whereas information on flu-related complications and high-risk pediatric populations is less common, highlighting a key area for future content development. It is also worth noting that nearly all content publishers express a positive stance toward influenza vaccination, with this tendency most pronounced on Douyin; while many BiliBili and Xiaohongshu publishers also create vaccine-related content, their overall tone toward vaccination is more neutral or negative by comparison.

Importantly, combining comment quantity and sentiment avoids the bias of single-dimensional metrics. For instance, BiliBili's low comment quantity (median = 0) could be misinterpreted as low engagement, but its relatively balanced sentiment distribution (21% positive, 15% negative) reveals that existing comments reflect genuine user interest in in-depth vaccine mechanisms rather than disengagement. Similarly, Xiaohongshu's moderate comment quantity (median = 3.5) paired with 19% positive sentiment indicates that its experiential content attracts targeted but less emotionally intense engagement. By integrating both dimensions of commenting behavior, this study offers a more nuanced understanding of how platform-specific content ecosystems shape user engagement with children's influenza vaccine information—and why commenting behavior serves as a superior proxy for engagement quality compared to isolated metrics like likes or views.

Short video platforms, therefore, need to enhance oversight and refine their algorithms to elevate the quality of health-related content. Publishers of videos should spotlight the benefits of flu vaccination—its safety, efficacy, and capacity to correct misinformation—while also underscoring how immunization reduces the risk, duration, and severity of influenza and protects vulnerable communities. Collaborative efforts must also leverage public health monitoring systems, expand health education in schools, and offer peer comparisons as well as online or electronic information services (16–18).

Across the GQS, JAMA, and mDISCERN scoring systems, videos created by healthcare professionals earned the highest marks, emphasizing their vital role in boosting content quality and building public trust. Conversely, those made by non-expert users scored lower, suggesting a need for greater professionalism and accuracy. News outlets and non-profits landed in the mid-range, signaling untapped potential in health communication. Moving forward, encouraging more healthcare professionals to produce content, guiding lay users to improve quality, fine-tuning news agencies' reporting accuracy, and strengthening non-profit engagement can all benefit from cross-sector collaboration. Such efforts will advance health-information dissemination, enrich health education, and continually raise the overall quality of health-related content.

## 5 Conclusions

While short videos offer insights into children's flu vaccines, their overall quality and reliability remain lacking. Most videos fall into a moderate range, revealing ample room for improvement through platform-specific strategies—such as amplifying healthcare professional content, addressing gaps in complications and high-risk population information, and integrating comment quantity and sentiment to refine engagement quality. To ensure the public gains access to accurate, actionable health knowledge, further refinements to these videos are essential.

## 6 Limitation

This study has several constraints. First, it focused solely on Douyin, BiliBili, and Xiaohongshu, leaving other platforms unexamined; their content quality also warrants investigation. Second, only one keyword was used during the video search, suggesting that future work should include additional terms to gather more comprehensive data. Finally, this study assessed only Chinese-language short videos, leaving the quality and reliability of content in other languages unaddressed.

## Data Availability

The raw data supporting the conclusions of this article will be made available by the authors, without undue reservation.
